# The Realization of One-to-Two-Port Beam Division in a Five-Channel Acoustic System

**DOI:** 10.3390/e27090949

**Published:** 2025-09-12

**Authors:** Rui Wang, Zhicheng Xu, Shuai Tang, Wencong Zhang, Jiabin Hou, Haipeng Cui, Yang Liu

**Affiliations:** 1 School of Navigation and Shipping, Shandong Jiaotong University, Weihai 264200, China; 2 Department of Research and Development, China Academy of Launch Vehicle Technology, Beijing 100076, China; 3 School of Information Science and Engineering, Harbin Institute of Technology, Weihai 264209, China; 4 Weihai Shuangxin Metal Products Co., Ltd., Weihai 264299, China; 5 School of Materials Science and Engineering, Harbin Institute of Technology, Weihai 264209, China; 6 School of Ocean Engineering, Harbin Institute of Technology, Weihai 264209, China; 7 Qingdao JARI Industrial Control Technology Co., Ltd., Qingdao 266000, China

**Keywords:** beam division, adiabatic transfer, acoustic system

## Abstract

In this work, one-to-two-port beam division is achieved in a five-channel acoustic system. The adjacent composing channels are connected by space-varying air slits, thus realizing quantum-like adiabatic energy transfer. Equal-weight beam splitting with opposite phases from two different output ports is obtained in a broadband signal of 6 kHz-10.5 kHz. In addition, owing to the existence of distinct evolution paths, one-way beam division is exhibited when a certain loss is evenly exerted inside the system. Furthermore, one-to-m-port beam division can also be achieved by extending the composing channels, thus making it possible to construct an asymmetric acoustic beam splitter. The simulated results verify that the incident waves can be split into opposite directions unidirectionally, which may have potential applications in concealed information transmission and eavesdropping.

## 1. Introduction

Owing to the advantages of miniaturization and integration, the realization of acoustic beam division in planar devices has numerous significant applications in fields such as communication, sensing, and detection. Although one-to-two-port beam division has been achieved in several previous works [1,2,3] by using acoustic couplers with Y-junctions, the robustness is relatively weak since the incident wavelength needs to match the channel size. Meanwhile, the configuration becomes complicated when increasing the output ports, and it is hard to obtain perfect performance in simple straight channels. Recently, an alternative approach has been proposed to obtain equal-weight beam division by utilizing the bandgaps or topological states in phononic crystals [4,5,6,7,8], which improves the stability of energy transfer behaviors. Nevertheless, a large number of scatterers with sophisticated shapes are required to be arranged along different directions, which unavoidably results in a bulky structural design [9,10,11,12,13]. Therefore, it is necessary to investigate an effective approach to realizing beam division in a simple acoustic system.

In past research, quantum-like adiabatic passage was adopted in the design of optical waveguide couplers, through which wave propagation behaviors can mimic the transfer actions of quantum states. A large number of attractive functions, such as frequency conversion [14], mode transformation [15], power absorption [16], energy exchange [17], and wave filtering [18], have been achieved in optical sokystems. The key factor to realize the modulation of energy transfer is to customize the space-dependent coupling strength between adjacent optical waveguides. Given the achievements of quantum-like adiabatic passage [19,20,21,22,23,24,25] in optical systems, functional waveguide couplers are also expected to be designed for acoustic systems. Different from the traditional design concept, quantum-like adiabatic passage offers an advanced approach to constructing a beam splitter by using straight channels instead of utilizing geometric variations in propagation paths.

As a remarkable achievement in quantum adiabatic passage, N. V. Vitanov [26] analyzed the extension of the three-state process of stimulated Raman adiabatic passage (STIRAP) to chainwise-connected multistate systems. A necessary condition for such a process is the existence of an adiabatic transfer state, which adiabatically connects the initial state of the chain to its final state. Based on this work, Ciret et al. [27] proposed a concept that exploits the evolution of an adiabatic transfer state in the system and leads to the multiple and achromatic splitting of light into *n* output ports. Inspired by the above works, we intend to adopt this idea in acoustics, through which a desired acoustic power splitter can be achieved. Meanwhile, we further intend to propose a novel approach to realizing asymmetric beam splitting in a free space by utilizing multi-port acoustic devices, which may have potential applications in concealed information transmission and eavesdropping.

Therefore, in this work, we propose a five-channel acoustically coupled system to achieve equal-weighted beam division. The system consists of straight channels connected by space-varying air slits along the direction of propagation. The acoustic waves incident from the left port of channel 1 can simultaneously transfer to the right ports of channels 3 and 5. Notably, the output waves from different ports not only exhibit equal intensity but also possess opposite phases. Due to the distinct evolution paths within the system, the energy transfer exhibits an asymmetric behavior when a specific loss is uniformly introduced in channel 2. This implies that the desired beam division occurs only for waves incident from the left port of channel 1. Furthermore, the one-to-*m*-port beam division can be achieved as well by extending the composing channels, offering a straightforward strategy for designing planar acoustic beam splitters.

## 2. Design and Methods

**Figure 1:**
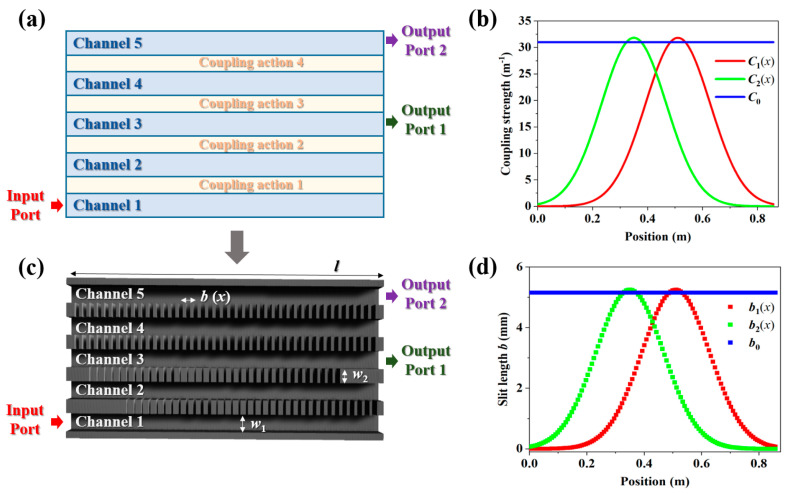
(**a**) A model of the five-channel acoustic coupler. (**b**) The required coupling strength between composing channels. (**c**) A schematic of the five-channel acoustic coupler. (**d**) The required air slit lengths between composing channels.

The model of the proposed five-channel acoustic coupler is shown in Figure 1a. The left side of channel 1 is selected as the input port, and the right sides of channels 3 and 5 are set as the output ports. The total length of the coupler is set as *L* = 0.875 m. The space-varying coupling action between channels 1 and 2 is *C*_1_(*x*), and that between channels 2 and 3 is *C*_2_(*x*). In actuality, the coupling actions *C*_1_(*x*) and *C*_2_(*x*) are the analogs to the Rabi frequencies for the pump and Stokes pulses in the STIRAP in quantum physics [28,29,30,31,32]. As a practical approach to simplifying the design framework, the coupling interactions among channels 3, 4, and 5 are spatially independent and constant. According to STIRAP theory [26], arrays with an odd number of channels support a dark state (adiabatic transfer state) provided the adiabatic condition is satisfied (see Appendix A).

Note that the existence of dark (adiabatic) states stems from destructive quantum interference and the symmetry inherent in the energy level structure of the system. For example, in one of the most common three-level Λ-type systems (odd number) used to generate dark states, the dark state can stably exist. By contrast, in the simplest two-level system (with an even number of levels), no dark state can be formed. A dark state refers to a state in which an atom cannot be excited to higher energy levels and therefore does not emit photons via spontaneous radiation. As a result, the atom appears “dark” or transparent to the laser field. The generation mechanism of the dark state is fundamentally due to quantum interference. When an atom attempts to transition from the dark state to an excited state, there are multiple possible pathways. The probability amplitudes (wave functions) of these pathways interfere destructively with each other, resulting in a net transition probability of zero.

In a three-level Λ-type system, there are two ground states |*g*_1_〉 and |*g*_2_〉 and one excited state |*e*〉. Laser 1 (with Rabi frequency Ω_1_) couples |g_1_〉 and |e〉, while laser 2 (with Rabi frequency Ω_2_) couples |*g*_2_〉 and |*e*〉. In this system, a special state can be identified—a superposition of the two ground states: |*D*〉 = (Ω_2_|*g*_1_〉 − Ω_1_|*g*_2_〉)/(Ω_1_^2^ + Ω_2_^2^)^1/2^. When attempting to apply the interaction Hamiltonian to |*D*〉, the result is zero. Physically, there are two pathways for the atom to transition from |*D*〉 to |*e*〉: one involves transitioning from the |*g*_1_〉 component to |*e*〉 with an amplitude proportional to +Ω_2_ * Ω_1_, while the other involves transitioning from the |*g*_2_〉 component to |*e*〉 with an amplitude proportional to − Ω_1_ * Ω_2_. These two pathways possess equal magnitude but opposite signs, leading to a complete destructive interference that cancels the total transition probability. As a result, atoms in the |*D*〉 state do not absorb photons and thus cannot be excited.

For a two-level system coupled by a laser between |*g*〉 and |*e*〉, any state can be expressed as |*ψ*〉 = *c_g_* |*g*〉 + *c_e_* |*e*〉. As long as the state contains an |*e*〉 component (*c_e_* ≠ 0), there is a possibility of photon absorption. As long as it contains a |*g*〉 component (*c_g_* ≠ 0), stimulated emission can occur. The transition from |*ψ*〉 to |*e*〉 has only one path (from the |*g*〉 component), and the transition from |*ψ*〉 to |*g*〉 also has only one path (from the |*e*〉 component). There is no second path to enable destructive interference. Therefore, in a two-level system, every state interacts with the light field, and no “dark” superposition state exists. To achieve destructive interference, at least three levels are required to provide two distinct transition paths. This is why three-level (odd-numbered) systems serve as the starting point for studying dark states.

When considering systems with more than three levels, the key factors are the topology of the energy level structure and the coupling methods. For a level chain |1〉 − |2〉 − |3〉 − …−|*N*〉, *N*-1 laser beams are used to couple adjacent levels sequentially (for example, laser 1 couples |1〉↔|2〉, laser 2 couples |2〉↔|3〉, and so on). In such a system, when *N* is odd, there always exists a dark state, as it can be viewed as a generalization of multiple Λ-type systems. The existence of a dark state is governed by the parity of the system. In an odd-numbered chain, a state can be constructed where the contributions of all excited state components coupled to the optical fields cancel each other out. This process is known as coherent population transfer, and its core is the dark state. In a simple even-numbered chain, there is typically no stable dark state that does not interact with all laser fields, since it is impossible to find a superposition where all possible excitation pathways perfectly cancel each other out.

It is anticipated that acoustic waves entering from the left port of channel 1 will propagate to the right ports of channels 3 and 5 with equal intensities and opposite phases. According to coupled mode theory, the wave propagation within the coupler can be described by

(1) $$i\frac{d}{dx}A=M(x)A.$$

To maintain generality, the coupling matrix *M*(*x*) of a coupled acoustic system with *N* + 2 (*N* = 1, 3, 5…) channels is expressed as

(2) $$M(x)=\left[\begin{array}{cccccc} 0 & C_{1}(x) & 0 & 0 & \ldots & 0\\ C_{1}(x) & 0 & C_{2}(x) & 0 & \ldots & 0\\ 0 & C_{2}(x) & 0 & C_{1,2} & \ldots & 0\\ 0 & 0 & C_{1,2} & 0 & \ddots & 0\\ \vdots & \vdots & & \ddots & \ddots & C_{N-1,N}\\ 0 & 0 & 0 & 0 & C_{N-1,N} & 0 \end{array}\right].$$

The amplitude of the modes in the individual channels is described as *A* = (*A_I_*, *A_B_*, *A*_1_, …, *A_N_*)^T^. The relative amplitude distribution at the output position (*x* = *L*) is

(3) $$\frac{A_{2k-1}}{A_{I}}={\left(-\right)}^{k}\frac{C_{1}(L)}{C_{2}(L)}\prod_{j=1}^{k-1}\frac{C_{2j-1,2j}}{C_{2j,2j+1}}={\left(-\right)}^{k}\frac{C_{1}(L)}{C_{2}(L)},$$

and *A*_2*k*_(*L*) = 0, and *A_B_*(*L*) = 0. The second equality in Equation (3) holds in our special case of a homogeneous channel array. When the coupling action *C*_1_(*L*) largely exceeds *C*_2_(*L*), no energy remains in channel 1. Consequently, if the input field is launched solely into channel 1, the output field is given by

(4) $$A(x=L)=\frac{1}{\sqrt{n}}{\left(0,0,-1,0,1,\ldots ,-1,0,1\right)}^{T},$$

where *n* = (*N* + 1)/2 is the number of excited output channels (number of output ports). In this work, we take *N* = 3 as an example (a five-channel acoustic system), and the total number of output channels is *n* = 2. As illustrated in Figure 1b, the coupling actions *C*_1_(*x*) (red line) and *C*_2_(*x*) (green line) are set as the Gaussian form: *C*_1_(*x*) = *C_m_*exp(−((*x* − *x*_0_) − *s*)^2^/*α*^2^), and *C*_2_(*x*) = *C_m_*exp(−((*x* − *x*_0_) + *s*)^2^/*α*^2^) with *α* = 0.167 m, *s* = 0.1 m, *C_m_* = 31.836 m^−1^, and *x*_0_ = 0.43 m. The coupling actions among straight channels 3, 4, and 5 are fixed as *C*_0_ = 31 m^−1^ (blue line). According to Equation (4), the output intensities in this case can be predicted as

(5) $$A^{2}(x=L)=\frac{1}{2}{\left(0,0,-1,0,1\right)}^{T}.$$

It is obtained from Equation (5) that an equal-weighted power splitting from channels 3 and 5 is achieved. Meanwhile, the output phase difference is π due to the existence of opposite signs between these two output channels.

The mapping of the designed Gaussian pulses into the parameter space, as shown in Figure 1c, is achieved by modulating the coupling actions between adjacent channels with space-varying air slits of different lengths *b*. The widths of the channels and slits are *w*_1_ = 10 mm and *w*_2_ = 8 mm, respectively. The considerable impedance mismatch between air and rigid materials results in much stronger coupling interactions for channels with high *b*-values than for those with low *b*-values. Hence, by scanning the parameter *b* at a certain frequency of *f* = 8.8 kHz, the relation between slit length and coupling strength is numerically calculated as *C* = *πb*/*a* with *a* being 518 mm^2^ (see Appendix B.). As shown in Figure 1d, the space-dependent slit lengths *b*_1_(*x*) and *b*_2_(*x*) and space-independent slit length *b*_0_ corresponding to coupling actions *C*_1_(*x*), *C*_2_(*x*), and *C*_0_ are plotted along the propagating direction.

## 3. Results and Discussions

**Figure 2:**
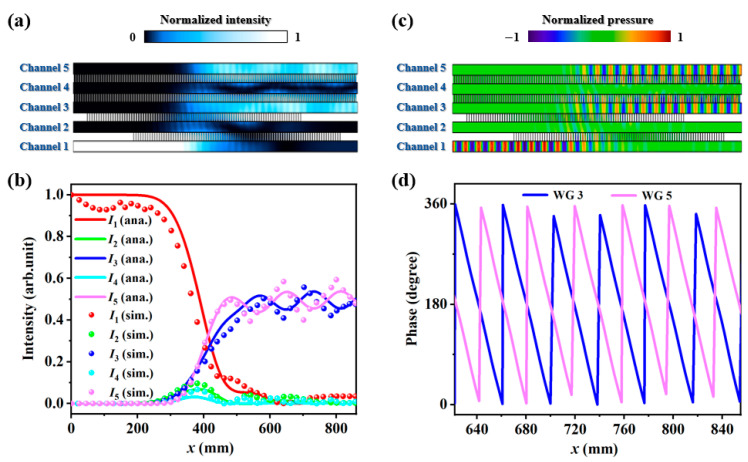
The acoustic (**a**) intensity and (**c**) pressure fields of the coupler for the left side of channel 1 incidence. The corresponding (**b**) intensity distributions and (**d**) phase distributions along the propagation direction. The colorful solid lines in (**b**) are the analytical results, and the colorful point lines are the corresponding simulated results.

Next, the beam division behavior of the coupler is validated through numerical simulations. The background medium for the five channels is set as air (velocity *c* = 343 m/s and density *ρ* = 1.21 kg/m^3^). To simplify the computational model, rigid materials are substituted with hard boundary conditions. Both sides of five channels are set as plane wave radiation conditions so as to avoid unwanted reflections. The working frequency is 8.8 kHz, and the incident pressure is 1 Pa. As shown in Figure 2a,b, the normalized intensities are localized in the left side of channel 1 at the initial position. After an adiabatic evolution process, the normalized intensities are equal-weighted in the right sides of channels 3 and 5 at the final positions, exhibiting a power splitting feature. In addition, as illustrated in Figure 2c,d, the output phases from channels 3 and 5 for such a one-to-two-port beam division are completely opposite, which agrees well with the theoretically predicted results in Equation (4). It is notable that the energy transfer efficiency remains relatively high, owing to the significantly low intensity distributions along channels 2 and 4 (see Appendix C). Moreover, if the coupling strength between channels 3 and 4 differs from that between channels 4 and 5, other power-splitting ratios can also be achieved.

In actuality, phase is a relative quantity, and its detection requires a reference point. Therefore, at least two sensors are required: one placed at the target detection point and the other placed at a fixed reference point. These two measurement microphones must be rigorously calibrated and exhibit closely matched frequency and phase responses. This is the foundation for obtaining accurate data; otherwise the phase difference in the instrument itself will contaminate the measurement results. In addition, a multi-channel data acquisition system is required to synchronously collect signals from two microphones. The specific operation steps are as follows:

I. Place the reference microphone (Reference Mic) at a fixed position within the waveguide (e.g., near the sound source or at the waveguide entrance).
II. Move the other probe microphone (Probe Mic) to the target location where we wish to measure the phase.
III. Simultaneously and synchronously record the time-domain signals (time waveforms) received by both microphones.
IV. Perform a Fourier transform (FFT) on both signals to convert them from the time domain to the frequency domain.
V. In the frequency domain, for the specific frequency of interest, extract the complex Fourier coefficients (containing magnitude and phase information) of the signals at both the probe point and the reference point.
VI. Calculate the phase difference between these two complex coefficients, which represents the relative phase of the probe point with respect to the reference point.

According to the above steps, the phase response of the expected position can be detected, and the phase difference between output ports can be measured.

**Figure 3:**
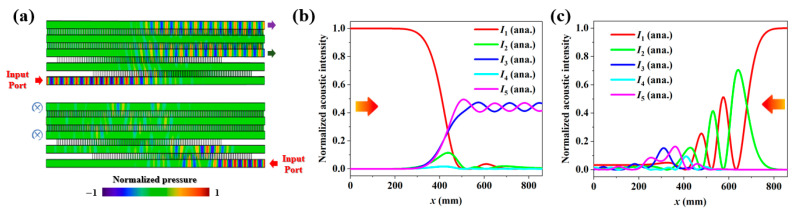
(**a**) The acoustic pressure fields of the five-channel coupler for the left port of channel 1 incidence and right port of channel 1 incidence, respectively. A certain loss is exerted inside channel 2. Normalized acoustic intensities along five channels for the (**b**) left port of channel 1 incidence and (**c**) right port of channel 1 incidence.

As mentioned before, two Gaussian pulses with a certain delay are adopted to design the coupling interactions of *C*_1_(*x*) and *C*_2_(*x*). Consequently, the multi-channel acoustic coupler has an asymmetric configuration along the propagating direction. For the left port of channel 1 incidence, the coupling action *C*_1_(*L*) largely exceeds *C*_2_(*L*), which is said to be the counterintuitive order in STIRAP. In contrast, for the right port of channel 1 incidence, the coupling action *C*_2_(*L*) is equivalent to significantly exceeding *C*_1_(*L*), which is said to be the intuitive order in STIRAP. In quantum STIRAP, these two scenarios yield distinct state transfer behaviors due to the presence of different evolution paths. For example, the population in the case of the intuitive order will strongly oscillate between quantum states, which can be ascribed to Rabi-like oscillations. As a counterpart, the propagating waves following quantum-like transfer behaviors will oscillate back and forth among composing channels for the right port of channel 1 incidence, displaying a huge difference for the case of the left port of channel 1 incidence. From Figure 2a,b, it is observed that the normalized intensities are mainly localized in channels 1, 3, and 5. Thus, as shown in Figure 3a, applying a uniform loss within channel 2 has minimal impact on the left port of channel 1 incidence, and a desired beam division is generated (Figure 3b). For the right port of channel 1 incidence, however, most of the input energies are dissipated inside the coupler, and the output intensities are relatively weak (Figure 3c). Therefore, it is concluded that equal-weighted beam splitting with opposite output phases is realized only in the case of the left port of channel 1 incidence, exhibiting an excellent one-way wave propagation feature.

To increase the number of output ports for beam division, more channels can be arrayed vertically. For example, a system with *N* = 5 employs seven air channels, thereby extending the number of output ports to *n* = 3. The coupling actions of *C*_1_(*x*) and *C*_2_(*x*) are implemented using the Gaussian pulses illustrated in Figure 1b, and the coupling actions among channels 3, 4, 5, 6, and 7 are fixed as *C*_0_ = 31 m^−1^. Following Equation (5), the output intensities in this case can be predicted as

(6) $$A(x=L)=\frac{1}{\sqrt{3}}{\left(0,0,-1,0,1,0,-1\right)}^{T}.$$

**Figure 4:**
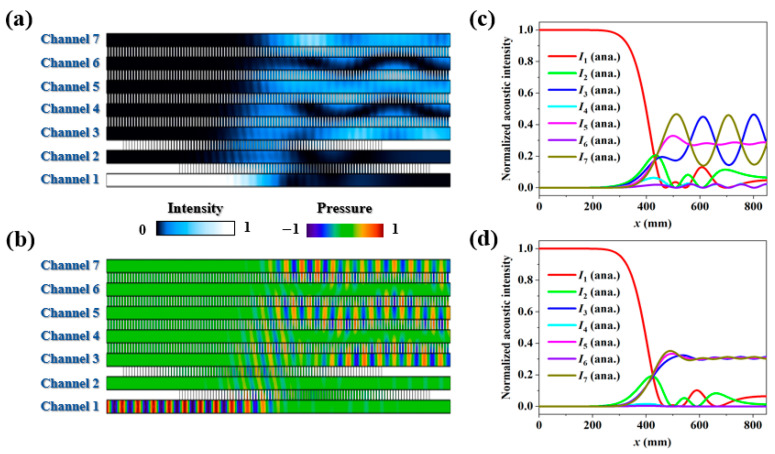
Acoustic (**a**) intensity and (**b**) pressure fields of seven-channel coupler for left port of channel 1 incidence. Normalized acoustic intensity distributions along seven channels in cases of (**c**) large value of *C*_0_ and (**d**) small value of *C*_0_.

As shown in Figure 4a,b, equal-weighted power splitting with opposite phases from channels 3, 5, and 7 is realized for the left port of channel 1 incidence. The normalized intensity distributions along the seven channels are plotted in Figure 4c. Although the output intensities from channels 3, 5, and 7 are nearly 0.33:0.33:0.33, an obvious oscillation is generated during the evolution process. This behavior is attributed to the fact that the coupling strength *C*_0_ in this case is too weak to ensure a smooth energy transfer behavior. By increasing the value of *C*_0_ to 60 m^−1^, as shown in Figure 4d, the oscillation can be effectively suppressed. Therefore, a stronger coupling interaction is required to stabilize the beam division process when extending the output ports.

**Figure 5:**
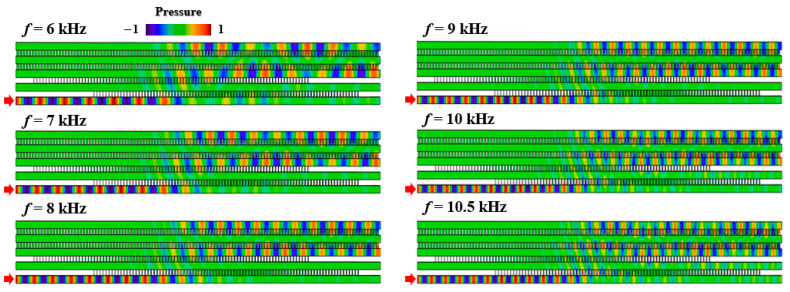
Acoustic pressure fields of five-channel coupler at 6 kHz, 7 kHz, 8 kHz, 9 kHz, 10 kHz, and 10.5 kHz.

As a unique advantage of the proposed beam splitter, the quantum-like adiabatic energy transfer behavior can exhibit robust performance. In previous works, to guide the propagating waves into different output ports, the designs of beam splitter always need sharp corners, leading to a narrow working band caused by the mismatch between working wavelength and corner size. As a comparison, the planar device proposed in this work consists of five straight channels without corners. Therefore, as illustrated in Figure 5, an expected beam division from two different output ports can be realized for the left port of channel 1 incidence in a broadband of 6–10.5 kHz, providing an alternative approach to designing robust acoustic devices.

**Figure 6:**
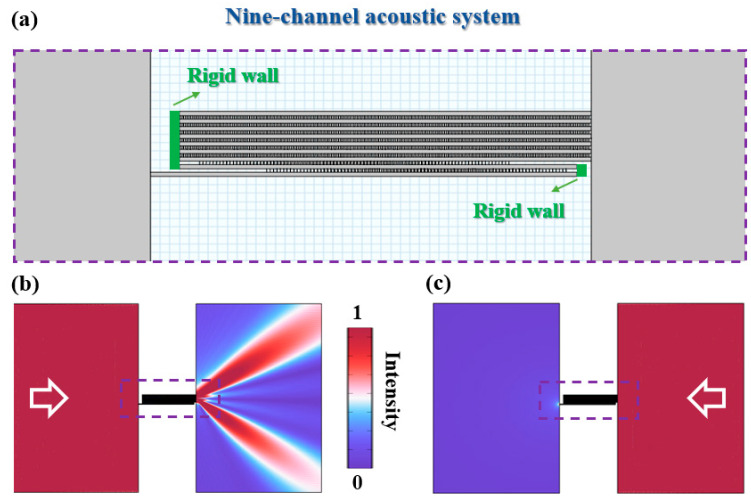
(**a**) A model of the asymmetric acoustic beam splitter based on a nine-channel acoustic system. Acoustic intensity fields for (**b**) left-side incidence and (**c**) right-side incidence at 9 kHz.

More uniquely, the realization of beam division with opposite phases provides an available approach to constructing an asymmetric acoustic beam splitter in a free space. Here, asymmetric transmission refers to the phenomenon where the same incident field is applied to the two sides of a device, but the outgoing behavior is different [33,34,35]. Taking a nine-channel acoustic system as an example, as shown in Figure 6a, the left ports of channels 2, 3, 4, 5, 6, 7, 8, and 9 and the right ports of channels 1 and 2 are sealed by rigid walls. Thus, the input port is the left side of channel 1, and the output ports are the right side of channels 3, 4, 5, 6, 7, 8, and 9 for left-side incidence. For right-side incidence, the situation is the opposite. Due to the asymmetric configuration of the device, the transmission fields are completely different in these two cases. For left-side incidence, as shown in Figure 6b, owing to the existence of the phase difference in π between adjacent odd cavities, the diffraction of a ±1-order wave is able to directly take one-pass propagation following the round-trip process demonstrated in [36,37], which is preferential to the 0-order wave, thus leading to a splitting beam in a free space. For right-side incidence, as shown in Figure 6c, the radiation field in a free space is relatively weak since the number of output ports is only one. Therefore, an asymmetric acoustic beam splitting device is realized based on the multi-channel coupled system, paving a new way for the design of functional acoustic devices.

## 4. Conclusions

In conclusion, an approach to the design of a planar acoustic beam splitter is shown in this work. The configuration of the device is only composed of five straight channels connected by space-dependent air slits. Equal-weighted beam splitting from two output ports of channels 3 and 5 is realized in a broadband of 6–10.5 kHz for the left port of channel 1 incidence, which follows the adiabatic passage in quantum systems. When uniform loss is introduced in channel 2, the beam division exhibits one-way energy transfer characteristics due to the presence of distinct evolution paths. Our design has the advantages of simple configuration, broadband response, and robust features, which may have potential applications in acoustic communication, sensing, and detection.

## Figures and Tables

**Figure A1:**
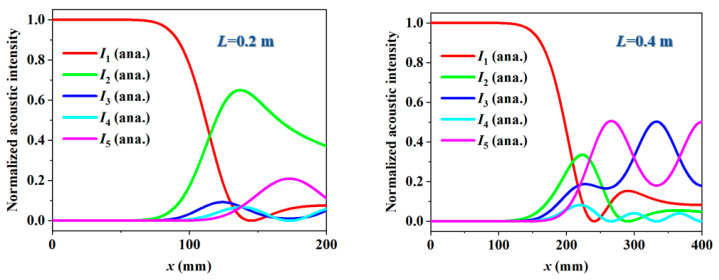
Normalized acoustic intensity distributions along five channels in cases of device length *L* = 0.2 m and *L* = 0.4 m.

**Figure A2:**
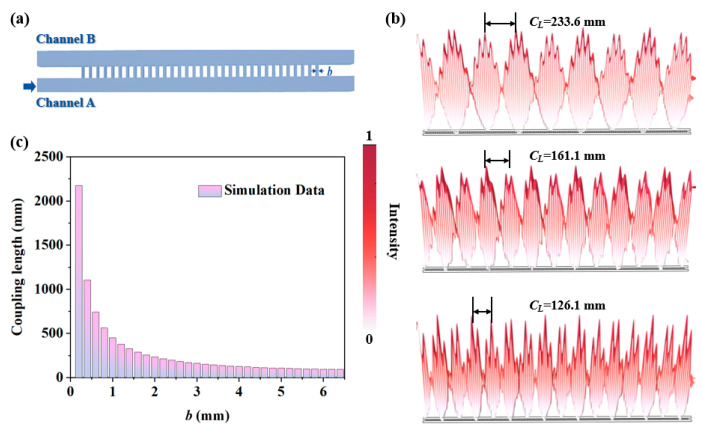
(**a**) Model of two-channel acoustic system. (**b**) Acoustic intensity distributions of two-channel system with different slit lengths. (**c**) Relation between slit length *b* and coupling length *C_L_*.

## Data Availability

Data will be made available on request.

## Appendix A. Discussions on Adiabatic Passage

For a channel chain |1〉 − |2〉 − |3〉−... −|*N*〉, *N*-1 coupling actions are used to couple adjacent channels sequentially. In such a system, when *N* is odd, there always exists a dark state, as it can be viewed as a generalization of multiple three-channel systems. Therefore, it is necessary to conduct a basic derivation of STIRAP in a simple three-channel acoustic system. The coupling matrix of the three-channel acoustic system is expressed as $M(x)=C_{1}(x)|2\rangle \langle 1|+C_{2}(x)|1\rangle \langle 2|+H.c.$, where $C_{1}(x)$ and $C_{2}(x)$ are the coupling actions between adjacent channels. For simplicity, the mixing angle is selected as $\theta \left(x\right)=arctan[C_{1}(x)/C_{2}(x)]$. The three eigenvalues of the system can be calculated as $\lambda_{\pm }=\pm \sqrt{{C_{1}\left(x\right)}^{2}+{C_{2}(x)}^{2}}$ and $\lambda_{0}=0$. The corresponding eigenstates are $\left|\left.B_{\pm }\left(x\right)\right\rangle \right.=\left[sin\theta \left(x\right)\left|\left.1\right\rangle \pm \left|\left.2\right\rangle +cos\theta \left(x\right)\left|\left.3\right\rangle \right.\right.\right.\right]/\sqrt{2}$ and $\left|\left.B_{0}\left(x\right)\right\rangle \right.=cos\theta \left(x\right)\left|\left.1\right\rangle -\right.sin\theta \left(x\right)\left|\left.3\right\rangle \right.$, respectively. Through the unitary transformation, the state function in the new basis vectors $\left\{\left|\left.B_{+}(x)\right\rangle \right.,\left|\left.B_{0}(x)\right\rangle \right.,\left|\left.B_{-}(x)\right\rangle \right.\right\}$ obeys the transformed Schrödinger-type equation:

(A1) $$i\frac{d}{dx}\psi (x)=[U^{†}M(x)U-iU^{†}\frac{dU}{dx}]\psi (x)$$

where we introduce a unitary transformation matrix:

(A2) $$U=\left(\begin{array}{ccc} \frac{\sin\theta (x)}{\sqrt{2}} & \cos\theta (x) & \frac{\sin\theta (x)}{\sqrt{2}}\\ \frac{1}{\sqrt{2}} & 0 & -\frac{1}{\sqrt{2}}\\ \frac{\cos\theta (x)}{\sqrt{2}} & -\sin\theta (x) & \frac{\cos\theta (x)}{\sqrt{2}} \end{array}\right).$$

Therefore, the systematic coupling matrix on the new basis vectors can be derived as

(A3) $$M_{a}(x)=U^{†}M(x)U-iU^{†}\frac{dU}{dx}=\left(\begin{array}{ccc} \varepsilon_{+}(x) & \frac{i\overset{\cdot }{\theta }(x)}{\sqrt{2}} & 0\\ -\frac{i\overset{\cdot }{\theta }(x)}{\sqrt{2}} & 0 & -\frac{i\overset{\cdot }{\theta }(x)}{\sqrt{2}}\\ 0 & \frac{i\overset{\cdot }{\theta }(x)}{\sqrt{2}} & -\varepsilon_{+}(x) \end{array}\right),$$

where $\varepsilon_{\pm }=\pm \sqrt{{C_{1}\left(x\right)}^{2}+{C_{2}(x)}^{2}}$. Following Equation (A3), it can be found that the off-diagonal terms of coupling matrix $M_{a}(x)$ can be ignored for a given condition (adiabatic condition).

(A4) $$\frac{1}{\sqrt{2}}\left|\overset{\cdot }{\theta }(x)\right|\ll \varepsilon_{+}(x).$$

To achieve efficient energy transfer behavior, the dark state $\left|\left.B_{0}\left(x\right)\right\rangle \right.=cos\theta \left(x\right)\left|\left.1\right\rangle -\right.sin\theta \left(x\right)\left|\left.3\right\rangle \right.$ can be selected when the adiabatic conditions are satisfied. For example, to realize a complete energy transfer from channel 1 to channel 3, the mixing angle $\theta \left(x\right)$ needs to change smoothly from 0 to *π*/2, which mimics the stimulated Raman adiabatic passage (STIRAP). As another example, the mixing angle $\theta \left(x\right)$ needs to change smoothly from 0 to *π*/4 if we intend to obtain equal-weighted power splitting from channel 1 to channels 1 and 3, which mimics the fractional stimulated Raman adiabatic passage (f-STIRAP). In systems with more than three channels, the dark state emerges provided that *N* is an odd number. This phenomenon arises from the fact that an *N*-channel system can be viewed as a generalization of a three-channel system.

To achieve the desired energy transfer behavior in acoustic systems, the adiabatic condition should be satisfied. Following Equation (A4), it is seen that the adiabatic condition can be fulfilled by the following approach: To obtain a small value of $\frac{1}{\sqrt{2}}|\dot{\theta }(x)|$, a long device length needs to be utilized to realize a slow-varying coupling strength. Therefore, the five-channel acoustic coupler designed in this work is relatively long (*L* = 0.875 m). Otherwise, as illustrated in Figure A1, the desired beam division cannot be realized when the device length is short (*L* = 0.2 m and *L* = 0.4 m).

## Appendix B. The Relation Between Coupling Strength and Slit Length

To map the quantum parameters (pump/Stokes pulses) to acoustic parameters (spatial coupling strength *C*_1_(*x*)/*C*_2_(*x*)), space-varying air slits with different lengths *b*(*x*) are utilized in this work. To explore the relation between the coupling strength and the length of air slit [*b*(*x*)], as shown in Figure A2a, we consider a simple two-channel acoustic coupled system. In this system, the coupling strength can be viewed as a constant C if the length of the connecting air slit [*b*(*x*)] between two composing channels is fixed, and the coupling matrix has the form of $M(x)=\left[\begin{array}{cc} 0 & C\\ C & 0 \end{array}\right]$. Supposing the pressure waveforms in channels A and B take the forms of $A\left(x\right)=a_{1}e^{-iqx}+a_{2}e^{iqx}$ and $B\left(x\right)=b_{1}e^{-iqx}+b_{2}e^{iqx}$, the following relations are deduced by solving the coupled mode equation:

(A5a) $$-iqa_{1}e^{-iqx}+iqa_{2}e^{iqx}=-iCb_{1}e^{-iqx}-iqb_{2}e^{iqx},$$

(A5b)
$$-iqb_{1}e^{-iqx}+iqb_{2}e^{iqx}=-iCa_{1}e^{-iqx}-iCa_{2}e^{iqx}.$$

From Equation (A5a,b), we can obtain q=C, $a_{1}=b_{1}$, and $a_{2}=b_{2}$. Combined with the initial conditions of $A\left(0\right)=a_{1}+a_{2}$ and $B\left(0\right)=b_{1}+b_{2}$, the pressure waveforms in channels A and B are further calculated as $A\left(x\right)=\cos\left(Cx\right)A\left(0\right)-isin(Cx)B(0)$ and $B\left(x\right)=\cos\left(Cx\right)B\left(0\right)-isin(Cx)B(0)$. If acoustic waves are illuminated from the left port of channel A, the initial conditions can be set as $A\left(0\right)=A_{0}$ and $B\left(0\right)=0$, and the acoustic power flows along the propagation direction of channels A and B are

(A6a) $$P_{A}(x)=\cos^{2}(Cx),$$

(A6b)
$$P_{B}(x)=1-\cos^{2}(Cx).$$

From Equation (A6a,b), it can be observed that the acoustic waves propagate back and forth between two composing channels, resulting in a periodic oscillation of intensity distribution. The distance between two adjacent peaks in the intensity field is defined as the coupling length $C_{L}$, which is inversely proportional to the coupling strength ($C_{L}=\pi /C$). From Figure A2b, we can find that a higher value of *b* leads to a smaller coupling length. By scanning the parameter *b* from 0.2 mm to 6.4 mm, as shown in Figure A2c, the relation between the coupling length and *b* can be well fitted by the function of $C_{L}=a/b$, where *a* is chosen to be 518 mm^2^. Then, the coupling strength can be further calculated by $C=\pi /C_{L}=\pi b/a$. Therefore, by changing the length of the connecting air slit [*b*(*x*)] between two composing channels, the coupling strength can be modulated flexibly so as to meet the space-varying requirement of the driven pulses.

## Appendix C. A Discussion of the Five-Channel Acoustic System

The coupling matrix of the five-channel acoustic system can be expressed as

(A7) $$M(x)=\left[\begin{array}{ccccc} 0 & C_{1}(x) & 0 & 0 & 0\\ C_{1}(x) & 0 & C_{2}(x) & 0 & 0\\ 0 & C_{2}(x) & 0 & C_{0} & 0\\ 0 & 0 & C_{0} & 0 & C_{0}\\ 0 & 0 & 0 & C_{0} & 0 \end{array}\right].$$

The evolution of the acoustic waves is determined by the coupled mode equation idA/dx=M(x)A [Equation (1)], where *A* = (*A*_1_, *A*_2_, *A*_3_, *A*_4_, *A*_5_)^T^ is the amplitude of the wave pressure in channel *k* (*k* = 1, 2, 3, 4, 5). The dark state of the proposed five-channel acoustic system is defined as the state that satisfies the condition of $M(x)\left|\left.B_{0}(x)\right\rangle \right.=0$. By solving the coupled mode equation, we can obtain *A*_2_ = 0, *A*_3_ + *A*_5_ = 0, *A*_4_ = 0 and *A*_1_ = −*A*_3_*C*_2_(*x*)/*C*_1_(*x*). Thus, the vector of the dark state can be expressed as

(A8) $$|B_{0}(x)\rangle =A_{3}{\left[\begin{array}{ccccc} -\frac{C_{2}(x)}{C_{1}(x)}, & 0, & 1, & 0, & -1 \end{array}\right]}^{T}$$

According to the coupling actions illustrated in Figure 1b, it is found that *C*_2_(*x*) >> *C*_1_(*x*) at the initial position, while *C*_2_(*x*) << *C*_1_(*x*) at the end position. Consequently, the normalized acoustic intensities are highly localized in channel 1 at the initial position (*x* = 0 m) owing to the fact that *C*_2_(*x*)/*C*_1_(*x*)→∞. Additionally, equal-weighted power splitting can be generated from channels 3 and 5 at the end position (*x* = 0.875 m) due to the fact that *C*_2_(*x*)/*C*_1_(*x*)→0. Therefore, by mapping the coupling actions *C*_1_(*x*) and *C*_2_(*x*) to space parameters *b*_1_(*x*) and *b*_2_(*x*), as shown in Figure 2a, the desired power splitting can be realized for the left port of channel 1 incidence. Meanwhile, as illustrated in Figure 2c, the output phases from channels 3 and 5 are opposite, which is attributed to the existence of opposite signs between *A*_3_ and *A*_5_ (−*A*_3_ = *A*_5_).

Through the above derivation, we clarified how the dark state condition suppresses output in channels 1, 2, and 4 while enabling channels 3 and 5 to function as an equal-weighted power splitter with opposite phases. This process relies on adiabatic variations in coupling strengths, which confine waves within the dark state throughout, thereby enabling efficient energy transfer. The entire system effectively extends the STIRAP principle to the design of multi-channel acoustic systems.
